# Home-Made Buttermilk

**Published:** 1907-11

**Authors:** 


### Home-Made Buttermilk

(Monthly Bulletin Indiana State Board of Health, June, 1907.)

It is now within the power of every household to have an abundance of that refreshing and healthful summer (also winter) drink—buttermilk. To the present time no one knew of any source of buttermilk except from the buttermaker; but nowadays the buttermaker does his work so well that the buttermilk is entirely deprived of the delicious little grains of fat which add so much to its food qualities as well as to taste. True buttermilk, made direct from fresh rich milk, within a few hours, of the finest flavor and taste, nutritious and more excellent than the article as originally known, can now be prepared in any kitchen. This is done by taking a quart of fresh, rich milk, adding a pinch of salt and about a half-pint of hot water to raise the temperature to body heat, and lastly adding a tablet which contains a pure culture of lactic acid bacteria. Place all in a pitcher, cover with a napkin, and let stand for twenty to twenty-four hours at the ordinary temperature, and there is your perfect buttermilk. The tablets are made by Parke, Davis & Co., pharmaceutical and chemical manufacturers, Detroit, Mich., and are called "lactone" or buttermilk tablets.

On the farm, in the process of buttermaking the cream is allowed to sour spontaneously and is then churned. The souring is the lactic acid fermentation caused by lactic acid bacteria or ferments. The difference between the new and old process is one of method and not result. In the old, the lactic fermentation is waited for and expected to occur spontaneously, with disappointment sometimes. In the new, the ferment in pure culture is directly planted in the milk, and the desired fermentation is secured without fail. In Bible days, spontaneous fermentation of dough was depended upon to leaven or lighten bread, and failure frequently attended the process, the dough putrefying instead of fermenting, and was then lost. Finally, man learned to add yeast to the dough and not to depend upon spontaneous processes, with the result of always securing the right fermentation and making a better and more nutritious bread. This new buttermilk process is a like improvement.

